# Neurodevelopment in Children Exposed to Antenatal CMV Viremia in a High HIV Prevalence Area

**DOI:** 10.1093/infdis/jiag214

**Published:** 2026-04-09

**Authors:** Ceri Evans, Jaya Chandna, Kuda Mutasa, Sandra Rukobo, Margaret Govha, Patience Mushayanembwa, Bernard Chasekwa, Florence D Majo, Gwendoline Kandawasvika, Batsirai Mutasa, Naume V Tavengwa, Robert Ntozini, Jean H Humphrey, Melissa J Gladstone, Andrew J Prendergast

**Affiliations:** Zvitambo Institute for Maternal and Child Health Research, Harare, Zimbabwe; Department of Clinical Infection, Microbiology and Immunology, University of Liverpool, Liverpool, United Kingdom; Centre for Genomics and Child Health, Blizard Institute, Queen Mary University of London, London, United Kingdom; Zvitambo Institute for Maternal and Child Health Research, Harare, Zimbabwe; Department of Infectious Disease Epidemiology and International Health, London School of Hygiene and Tropical Medicine, London, United Kingdom; Zvitambo Institute for Maternal and Child Health Research, Harare, Zimbabwe; Zvitambo Institute for Maternal and Child Health Research, Harare, Zimbabwe; Zvitambo Institute for Maternal and Child Health Research, Harare, Zimbabwe; Zvitambo Institute for Maternal and Child Health Research, Harare, Zimbabwe; Zvitambo Institute for Maternal and Child Health Research, Harare, Zimbabwe; Zvitambo Institute for Maternal and Child Health Research, Harare, Zimbabwe; Faculty of Medicine and Health Sciences, University of Zimbabwe, Harare, Zimbabwe; Zvitambo Institute for Maternal and Child Health Research, Harare, Zimbabwe; Zvitambo Institute for Maternal and Child Health Research, Harare, Zimbabwe; Zvitambo Institute for Maternal and Child Health Research, Harare, Zimbabwe; Zvitambo Institute for Maternal and Child Health Research, Harare, Zimbabwe; Department of International Health, Johns Hopkins Bloomberg School of Public Health, Johns Hopkins University, Baltimore, Maryland, USA; Department of Women's & Children's Health, University of Liverpool, Liverpool, United Kingdom; Zvitambo Institute for Maternal and Child Health Research, Harare, Zimbabwe; Centre for Genomics and Child Health, Blizard Institute, Queen Mary University of London, London, United Kingdom

**Keywords:** Africa, HIV-exposed uninfected, co-infection, CMV, pregnancy

## Abstract

We compared neurodevelopment at age 24 months among children born to mothers with or without cytomegalovirus (CMV) viremia in the third trimester of pregnancy in rural Zimbabwe, using the Malawi Developmental Assessment Tool (MDAT) for gross motor, fine motor, language and social development. Among 209 children, 49 (23%) were exposed to third-trimester CMV viremia. The mean total MDAT score was 91.0 (SD 9.4) in CMV-exposed children versus 94.1 (8.8) in CMV-unexposed children (adjusted difference −4.02 points [95% CI −7.01, −1.04, *P* = .008]). Cytomegalovirus exposure in utero, without evidence of congenital infection, may impair child neurodevelopment, providing a potential antenatal intervention target.

## Teaser

Antenatal exposure to cytomegalovirus (CMV) may be one factor that reduces neurodevelopmental potential in sub-Saharan Africa. Cytomegalovirus frequently reactivates during pregnancy, particularly in the context of maternal HIV infection; this finding may therefore have important public health implications.

In sub-Saharan Africa, two-thirds of children fail to reach their developmental potential [1]. Early-life exposures shape neurodevelopment in the first 1000 days, from conception to 2 years, which is a period of rapid and intricately-coordinated brain development. We previously found that children in rural Zimbabwe with HIV-1 exposure had delayed neurodevelopment at age 2 years compared with children without HIV-1 exposure, even when they remained HIV-free themselves [2]. The reasons remain uncertain, but exposure to other co-infections may contribute [3].

Cytomegalovirus (CMV) is almost ubiquitously acquired during childhood in sub-Saharan Africa [4], and reactivation can occur during pregnancy, particularly in women living with HIV [5]. We hypothesized that exposure to maternal CMV in utero may affect neurodevelopment in settings of high HIV prevalence. Here, we evaluated neurodevelopment among children born to mothers with and without CMV viremia in the third trimester of pregnancy in rural Zimbabwe.

## METHODS

### Study Population

The SHINE trial (ClinicalTrials.gov/NCT01824940) enrolled Zimbabwean women during pregnancy to assess the effects of infant and young child feeding (IYCF) and water, sanitation, and hygiene (WASH) interventions on child stunting, anemia, and neurodevelopment [6–9]. The Medical Research Council of Zimbabwe (MRCZ/A/1675) and Johns Hopkins Bloomberg School of Public Health (JHU IRB #4205) approved the protocol. All mothers provided written informed consent.

### Data Collection

Research nurses collected maternal data and blood during pregnancy at median 14 and 32 gestational weeks. Infants were seen at 1, 3, 6, 12, and 18 months postpartum, with plasma and saliva collection in a subgroup. Mother–infant pairs were tested longitudinally for HIV, as previously described [10]. To measure maternal HIV viral loads, viral nucleic acid was extracted from 200μL of cryopreserved plasma collected at baseline, using the Abbott RealTime HIV-1 extraction assay, followed by real-time PCR on the Abbott m2000rt platform using Abbott RealTime HIV Amplification Reagents (limit of detection 150 copies/mL) [11].

### CMV Testing

Cytomegalovirus was measured at 32 gestational weeks in all women with HIV who had available cryopreserved plasma (N = 358 mothers of 364 children), and a random sample of women without HIV (N = 85 mothers of 87 children). Viral nucleic acid was extracted from 100 μL plasma using the QIAamp DSP Virus Spin Kit. Cytomegalovirus was detected by real-time PCR on the Abbott m2000rt platform using Abbott RealTime CMV Amplification Reagents, modified for the extraction method used, providing a limit of quantification of 45 copies/mL. Each run included positive and negative controls. For infants, viral nucleic acid was extracted from 50 μL of saliva, collected at 3 months of age, using the same methods, with a limit of quantification of 90 copies/mL; positive saliva samples <450 copies/mL were considered negative, as previously described [11]. To limit false-positive results from maternal breast milk residue, infant saliva was collected at least 20 minutes after breastfeeding. We were unable to distinguish congenital versus acquired CMV infection because samples were not collected in the first 3 weeks after birth; among those testing CMV-negative at 3 months of age, we reasoned that congenital CMV was unlikely because CMV DNA would still be detectable at 3 months [12].

### Neurodevelopment Substudy

Children who turned 2 years old (allowable age-window 102–112 weeks) between March 2016-April 2017 were eligible for the neurodevelopment substudy [6, 7]. Research nurses, who underwent 3 weeks of residential training and regular standardization, used the Malawi Developmental Assessment Tool (MDAT) to evaluate child development in 4 domains (gross motor [36 points], fine motor [36], language [36], social [30]; total 138 points) [13]. The fine motor, language and social domains also measure components of cognitive development.

### Statistical Analyses

We compared baseline characteristics between CMV-exposed and CMV-unexposed children while handling within-cluster correlation using multinomial and ordinal regression models with robust variance estimation, and Somers’ D for medians. We compared MDAT scores between children born to mothers with versus without detectable CMV viremia at 32 gestational weeks using generalized estimating equations (GEEs) with an independent working correlation structure. Three multivariable models were built, adjusted for trial arm (Model 1); study-related factors (arm, study nurse undertaking neurodevelopmental testing, infant age, month of birth, maternal HIV; Model 2); and study-related factors plus potential socioeconomic and demographic cofounders (arm, study nurse, infant age, month of birth, maternal HIV, age, education, employment, parity, wealth [14], and infant birthweight and sex; Model 3). In a sensitivity analysis, we evaluated associations between maternal CMV and child MDAT scores among children confirmed CMV-negative at 3 months. Subgroup analyses were undertaken if there was evidence of interaction (*P* < .05, Wald test) between maternal HIV and CMV viremia; between detectable HIV viremia at the enrollment into the trial (median 14 gestational weeks, as a marker of early ART) and CMV viremia; or between infant sex and CMV viremia. Subgroup analyses in the HIV-exposed group were also adjusted for maternal HIV disease severity (viral load/CD4 count). All sensitivity and subgroup analyses were fully adjusted for the same covariates (Model 3). Analyses were carried out using Stata version 18.0.

## RESULTS

Among 5280 enrolled pregnant women, there were 4727 live births; 738 children were HIV-exposed and 3989 children were HIV-unexposed. Three hundred and twenty-three and 1655 children who were HIV-exposed and HIV-unexposed, respectively, were enrolled in the ECD substudy. Children were subsequently excluded if they were outside the predefined age window (N = 28), had severe disability (N = 17), tested HIV-positive (N = 6), or had unknown HIV status (N = 12). Of the remaining 300 and 1615 HIV-exposed and HIV-unexposed children, 40 and 169 children were born to women who had CMV testing in the third trimester (Supplementary Figure 1). Overall, 49 children with intrauterine CMV exposure and 160 children without CMV exposure were included in the analysis.

### Baseline Characteristics

Baseline characteristics of mothers, households, and children are shown in Supplementary Table 1. Women with CMV viremia in the third trimester compared with those without CMV viremia were more likely to be living with HIV (88% vs 79%; *P* = .07). Forty-three (27%) of 169 children born to mothers with HIV had CMV exposure compared with 6 (15%) of 40 children without HIV exposure (odds ratio 1.93, 95% CI .76, 4.92). Birthweight of children with versus without CMV exposure tended to be lower (mean [SD] 2.94 [0.5] versus 3.08 [0.5] kg, respectively; *P* = .06).

### Neurodevelopmental Outcomes

Neurodevelopmental outcomes in children with and without intrauterine CMV exposure are shown in Table 1. Mean (SD) total MDAT score was 91.0 (9.4) in children with CMV exposure compared to 94.1 (8.8) in children without CMV exposure. The fully adjusted difference was −4.02 points (95% confidence interval (CI) −7.01, −1.04, *P* = 0.008), which is equivalent to ∼0.4 SD difference in total MDAT score between groups. Within the MDAT assessment, the findings were driven mostly by differences in fine motor (−1.67, 95%CI −2.83, −0.52, *P* = 0.004) and language scores (−1.80, 95%CI −3.04, −0.56, *P* = 0.005). There was no evidence of differences in gross motor (−0.15, 95%CI −1.21, 0.90, *P* = 0.778) or social (−0.40, 95%CI −1.11, 0.31, *P* = 0.269) scores (Table 1). There was no difference in mean head circumference-for-age Z-scores at 18-months (−0.47 (SD 1.04) vs −0.29 (1.11); *P* = 0.58).

**Table 1.** Associations Between CMV Exposure and MDAT Scores

	Mean (SD)		Mean Difference (95%CI)					
Third Trimester CMV Exposure	Yes N = 49	No N = 160	Model 1^a^	*P*	Model 2^b^	*P*	Model 3^c^	*P*
Total MDAT score, mean (SD)	91.0 (9.4)	94.1 (8.8)	−3.30 (−6.41, −0.20)	.037	−3.69 (−6.67, −0.71)	0.015	−4.02 (−7.01, −1.04)	.008
Gross motor, mean (SD)	23.8 (2.9)	23.7 (3.3)	−0.12 (−1.14, 0.91)	.822	−0.14 (−1.20, 0.92)	.796	−0.15 (−1.21, 0.90)	.778
Fine motor, mean (SD)	22.4 (4.1)	23.6 (2.5)	−1.22 (−2.37, −0.06)	.039	−1.52 (−2.68, −0.36)	.011	−1.67 (−2.83, −0.52)	.004
Language, mean (SD)	20.6 (4.1)	21.9 (4.1)	−1.52 (−2.89, −0.16)	.029	−1.61 (−2.83, −0.39)	.010	−1.80 (−3.04, −0.56)	.005
Social, mean (SD)	24.3 (2.2)	24.7 (2.2)	−0.45 (−1.22, 0.33)	.257	−0.42 (−1.10, 0.26)	.231	−0.40 (−1.11, 0.31)	.269

^a^Regression models adjusted for randomized trial arm.

^b^Regression models adjusted for study-related factors in addition to randomized trial arm: neurodevelopmental assessor at 24-month visit, exact infant age at 24-month visit, sex, calendar month of birth, and maternal HIV status.

^c^Regression models adjusted for all variables in Model 2 plus baseline covariates that may be associated with CMV exposure (birthweight; maternal age, education, parity and employment status; and household wealth).

#### Sensitivity and Subgroup Analyses

By age 3 months, 55 (57%) of 97 children with saliva samples were CMV-positive and 42 (43%) were CMV-negative. Restricting analyses to children testing CMV-negative at 3 months, findings were similar (mean [SD] total MDAT scores 89.0 [11.5] in those with antenatal CMV exposure [N = 5] versus 94.1 [7.4] in those without CMV exposure [N = 37]; adjusted difference −8.01 [95% CI −12.62, −3.56; *P* < .001]), suggesting effects were unlikely driven by more children with congenital CMV in the exposed group.

There was a significant interaction with HIV exposure status (*P* = .04); we therefore stratified findings by maternal HIV status. There were associations between MDAT scores and CMV exposure status in both HIV-exposed and HIV-unexposed children, although effect sizes tended to be greater in children born to women without HIV (Table 2). When HIV-exposed children were stratified by maternal HIV viral load (detectable vs undetectable, interaction *P* = .01), associations between CMV exposure and MDAT were found in those born to mothers with undetectable HIV viral load but not those exposed to detectable HIV viremia (Table 2). Child sex did not modify the effects of CMV exposure on MDAT score (interaction *P* = .53).

**Table 2.** Associations Between CMV Exposure and MDAT Scores According to HIV Exposure

	Infant HIV Exposure Status	Third Trimester CMV Exposure		Adjusted Difference (95% CI)^a^	*P*
		Yes	No		
Total score Mean (SD)	HIV-exposed	91.8 (9.4)	93.8 (9.1)	−3.19 (−6.30, −0.08)	.045
	Detectable HIV viral load	95.0 (10.5)	94.0 (9.2)	−2.06 (−8.59, 4.47)	.536
	Undetectable HIV viral load	87.4 (5.4)	94.2 (9.4)	−7.39 (−10.79, −3.99)	<.001
	HIV-unexposed	85.7 (8.5)	94.9 (7.8)	−4.58 (−8.35, −0.80)	.017
Gross motor Mean (SD)	HIV-exposed	23.8 (3.0)	23.8 (3.5)	−0.04 (−1.23, 1.16)	.953
	Detectable HIV viral load	24.8 (3.4)	23.6 (3.5)	0.59 (−1.56, 2.74)	.588
	Undetectable HIV viral load	22.8 (1.9)	24.4 (3.6)	−1.91 (−3.29, −0.53)	.007
	HIV-unexposed	23.5 (1.6)	23.6 (2.6)	0.39 (−1.35, 2.13)	.660
Fine motor Mean (SD)	HIV-exposed	22.7 (4.0)	23.5 (2.6)	−1.37 (−2.70, −0.05)	.043
	Detectable HIV viral load	22.8 (4.8)	23.9 (2.2)	−1.90 (−4.25, 0.44)	.112
	Undetectable HIV viral load	22.6 (2.8)	23.4 (3.0)	−1.16 (−2.98, 0.66)	.213
	HIV-unexposed	20.3 (4.0)	24.0 (1.9)	−2.55 (−3.92, −1.17)	<0.001
Language Mean (SD)	HIV-exposed	20.9 (4.2)	21.7 (4.0)	−1.48 (−2.72, −0.23)	.021
	Detectable HIV viral load	22.7 (4.5)	21.8 (4.4)	−0.42 (−2.95, 2.11)	.746
	Undetectable HIV viral load	18.4 (1.9)	21.7 (3.8)	−3.30 (−4.49, −2.12)	<.001
	HIV-unexposed	18.2 (2.3)	22.8 (4.1)	−2.11 (−4.14, −0.09)	.041
Social Mean (SD)	HIV-exposed	24.3 (2.2)	24.8 (2.2)	−0.31 (−1.09, 0.47)	.441
	Detectable HIV viral load	24.7 (2.1)	24.7 (2.1)	−0.33 (−1.46, 0.80)	.565
	Undetectable HIV viral load	23.6 (2.5)	24.8 (2.3)	−1.02 (−2.28, 0.25)	.116
	HIV-unexposed	23.7 (1.6)	24.5 (2.3)	−0.31 (−1.92, 1.30)	.708

^a^Regression models adjusted for randomized trial arm, infant month of birth, sex and birthweight, exact infant age at 24-month visit, neurodevelopmental assessor at 24-month visit, and baseline maternal factors (maternal age, education, parity, employment status and household wealth, and CD4 count and use of antiretroviral therapy in women living with HIV). Detectable HIV viral load was defined as a viral load ≥150 copies/mL.

## DISCUSSION

In a rural Zimbabwean birth cohort, we found an association between CMV viremia in late pregnancy and child neurodevelopment at 2 years. After adjusting for potential confounders, the strongest evidence of difference between CMV-exposed and CMV-unexposed children was in 2 cognitive domains: fine motor coordination and language development. Exposure to CMV viremia in utero, without congenital CMV infection, may therefore be associated with neurodevelopmental delay in sub-Saharan Africa, highlighting a potentially important pathway, which requires further study.

This study was conducted in a high antenatal HIV prevalence setting, and children who were HIV-exposed but uninfected were deliberately oversampled because we previously reported that HIV exposure was associated with impaired neurodevelopment [2] and CMV viremia is common in pregnant women with HIV. However, associations between CMV and MDAT scores were found in children born to mothers both with and without HIV. Women had similar baseline characteristics regardless of CMV viremia detection, and analyses were adjusted for potential confounders, meaning CMV exposure was not merely a proxy for HIV, disease severity, low socioeconomic status, or education level.

Although women with HIV may be more likely to have CMV viremia than women without HIV, associations between CMV and MDAT scores were actually of greater magnitude in children who were either HIV-unexposed or born to mothers with well-controlled HIV (undetectable viral loads at trial recruitment), compared with those who were exposed to maternal HIV viremia. Small numbers in subgroup analyses limit firm conclusions, but it is plausible that CMV has relatively less impact when exposure to a second infection affecting neurodevelopment (ie, active HIV viremia) is also present. Differences in MDAT scores between CMV-exposed and CMV-unexposed children were larger than the magnitude of difference we previously reported between HIV-exposed and HIV-unexposed children (0.4 vs 0.15 standard deviations) [2]. Both HIV and CMV exposure were associated with reduced language development, but it is interesting that the main contributor to the MDAT difference by HIV-exposure in this same cohort was gross motor development [2]—a domain not associated with CMV in this substudy. The fine motor and language domains both assess cognitive development, meaning this may be the aspect of neurodevelopment most impacted by CMV exposure. The potential mechanisms through which CMV could affect neurodevelopment include *directly* infecting the fetal central nervous system, leading to disruption of neuronal migration and synapse formation, or *indirectly* through placental inflammation and subsequent interruption of oxygen and nutrient supply [15]. Direct infection would lead to congenital CMV, so an indirect mechanism via placental pathology is a more likely pathway for CMV exposure without vertical transmission.

This study has limitations. We sampled a small proportion of children from the SHINE trial, which may have introduced bias; in particular, few children included here were born to mothers without HIV. Nevertheless, sociodemographic factors were similar to the whole trial population, and this rural birth cohort had longitudinal sampling, direct neurodevelopmental assessments, and rich covariate data. Laboratory analyses relied on sample collection, availability, and storage. We were unable to diagnose congenital CMV, which required samples collected closer to birth; however, in a sensitivity analysis restricted to children testing CMV-negative at 3 months, findings were similar. In addition, we focused on third trimester CMV viremia, rather than exploring associations with earlier viremia, which could have led to different results. We measured CMV in only one compartment (plasma), possibly underestimating the degree of CMV reactivation during pregnancy. We were only able to assess associations, rather than causality; we constructed multivariable models to account for covariates, but there may be residual confounding.

In summary, antenatal exposure to CMV may be one factor that reduces neurodevelopmental potential in sub-Saharan Africa. By adulthood, CMV is almost ubiquitous in sub-Saharan Africa, and CMV frequently reactivates during pregnancy, particularly in the context of maternal HIV infection; this finding may therefore have important public health implications. Although we were unable to assign causality to the relationship, detection of CMV viremia and CMV viral suppression during pregnancy, particularly in HIV-CMV co-infection, may be a plausible strategy to help ensure that all children thrive.

## Supplementary Material

jiag214_Supplementary_Data

## Supplementary Data

Supplementary materials are available at *The Journal of Infectious Diseases* online (http://jid.oxfordjournals.org/). Supplementary materials consist of data provided by the author that are published to benefit the reader. The posted materials are not copyedited. The contents of all supplementary data are the sole responsibility of the authors. Questions or messages regarding errors should be addressed to the author.
