# Supplementary material for: Neurodevelopment in Children Exposed to Antenatal CMV Viremia in a High HIV Prevalence Area
Source: J Infect Dis. 2026 Apr 9;234(3):e595–9. doi: 10.1093/infdis/jiag214 (PMC13600408; doi:10.1093/infdis/jiag214)
Supplement: Supplementary file 1 [file jiag214_supplementary_data.docx]

**Figure 1. Flow of participants through trial**

**1** woman enrolled once for two pregnancies in error

**11** women enrolled twice in error

**5280** pregnant women enrolled from 210 randomized clusters^1^

**5270** pregnant women enrolled from 211 randomized clusters^1^

**82** additional fetuses

**49** women exited

**90** lost to follow up antenatally

**252** miscarriages

**113** stillbirths

**4** maternal deaths

**116** fetuses of **114** HIV-unknown mothers

**738** live infants born to **726** HIV-positive mothers

**3989** live infants born to **3937** HIV-negative mothers

**198** infant deaths

**100** lost to follow up

**5** exits

**51** infant deaths

**18** lost to follow up

**1** exit

**18 months**

**3686** HIV-unexposed children assessed for trial primary outcome

**18 months**

**668** HIV-exposed children assessed for trial primary outcome

**1085** not eligible for ECD sub-study

**193** not eligible for ECD sub-study

**2601** HIV-unexposed children eligible for ECD sub-study

**475** HIV-exposed children eligible for ECD sub-study

**946** not enrolled

**152** not enrolled

**1655** HIV-unexposed children enrolled into ECD sub-study

**323** HIV-exposed children enrolled into ECD sub-study

**3** outside age range

**2** severe disability

**6** HIV-positive

**12** HIV-unknown

**25** outside age range

**15** severe disability

**300** HIV-exposed uninfected children evaluated in ECD sub-study

**1615** HIV-unexposed children evaluated in ECD sub-study

**1575** no maternal sample tested for CMV in third trimester

**131** no maternal sample tested for CMV in third trimester

**169 HIV-exposed uninfected children included in final analysis**

**40 HIV-unexposed children included in final analysis**

Supplementary Table 1. Maternal, household, and infant baseline characteristics.

| **Baseline characteristics^1^** | **CMV-exposed** | **CMV-unexposed** | ***P*** |
| --- | --- | --- | --- |
| Mothers [N] | 49 | 156 |  |
| Infants [N] | 49 | 160 |  |
| **Trial arm^2^**  Standard of care  IYCF  WASH  IYCF+WASH  **Household characteristics** | 12/49 (25%)  14/49 (29%)  5/49 (10%)  18/49 (37%) | 24/160 (15%)  36/160 (23%)  41/160 (26%)  59/160 (37%) | 0.04 |
| Wealth quintile: |  |  | 0.97 |
| Lowest | 13 (27%) | 39 (25%) |  |
| Second | 10 (21%) | 32 (21%) |  |
| Middle | 9 (19%) | 28 (18%) |  |
| Fourth | 10 (21%) | 29 (19%) |  |
| Highest | 6 (13%) | 26 (17%) |  |
| **Maternal characteristics** |  |  |  |
| Age, years; mean (SD) | 30.4 (6.5) | 29.5 (6.1) | 0.34 |
| Height, cm; mean (SD) | 160.0 (6.0) | 160.2 (6.7) | 0.92 |
| MUAC, cm; mean (SD) | 26.6 (3.4) | 26.5 (2.9) | 0.79 |
| Completed schooling, years; mean (SD) | 9.4 (1.6) | 9.2 (1.9) | 0.50 |
| Married | 42/45 (93%) | 140/146 (96%) | 0.83 |
| Employed | 1/47 (2%) | 15/154 (10%) | 0.15 |
| Religion: |  |  | 0.81 |
| Apostolic | 21/49 (43%) | 75/156 (48%) |  |
| Other Christian religions | 21/49 (43%) | 63/156 (40%) |  |
| Other non-Christian religions | 7/49 (14%) | 18/156 (12%) |  |
| Living with HIV | 43/49 (88%) | 123/156 (79%) | 0.07 |
| **Infant characteristics** |  |  |  |
| Female | 21/49 (43%) | 82/160 (51%) | 0.26 |
| Birth weight, kg; mean (SD) | 2.94 (0.5) | 3.08 (0.5) | 0.06 |
| Birth weight <2.5kg | 6/48 (13%) | 17/154 (11%) | 0.20 |

^1^ Baseline for mothers was 2 weeks after consent (~14 weeks gestation). Baseline for infants was at birth. Values are %, unless stated.

^2^ IYCF = infant and young child feeding; WASH = water, sanitation and hygiene.

SD: standard deviation; IQR: interquartile range; MUAC: mid-upper arm circumference.
